# Supplementary material for: Modeling the Orthosteric Binding Site of the G Protein-Coupled Odorant Receptor OR5K1
Source: J Chem Inf Model. 2023 Jan 25;63(7):2014–29. doi: 10.1021/acs.jcim.2c00752 (PMC10091413; doi:10.1021/acs.jcim.2c00752)
Supplement: Supplementary file 1 — ci2c00752_si_001.pdf [file ci2c00752_si_001.pdf]

Supporting Information

Modeling the Orthosteric Binding Site of the G Protein-Coupled Odorant Receptor OR5K1

Alessandro Nicoli,[1‡] Franziska Haag,[1‡] Patrick Marcinek,[1] Ruiming He,[1,2] Johanna Kreißl,[1] Jörg Stein,[1] Alessandro Marchetto,[3,4] Andreas Dunkel,[1] Thomas Hofmann,[5] Dietmar Krautwurst,[1] Antonella Di Pizio[1*]*

1 Leibniz Institute for Food Systems Biology at the Technical University of Munich, 85354 Freising, Germany

2 Department of Chemistry, Technical University of Munich, D-85748 Garching, Germany

3 Computational Biomedicine, Institute for Advanced Simulations (IAS)-5/Institute for Neuroscience and Medicine (INM)-9, Forschungszentrum Jülich, 52428 Jülich, Germany

4 Department of Biology, Faculty of Mathematics, Computer Science and Natural Sciences, RWTH Aachen University, 52074 Aachen, Germany

5 Chair of Food Chemistry and Molecular Sensory Science, Technical University of Munich, 85354 Freising, Germany

* Correspondence: a.dipizio.leibniz-lsb@tum.de; Tel.: +49 8161716516, d.krautwurst.leibniz-lsb@tum.de; Tel.: +49 8161712634

‡ These authors contributed equally.

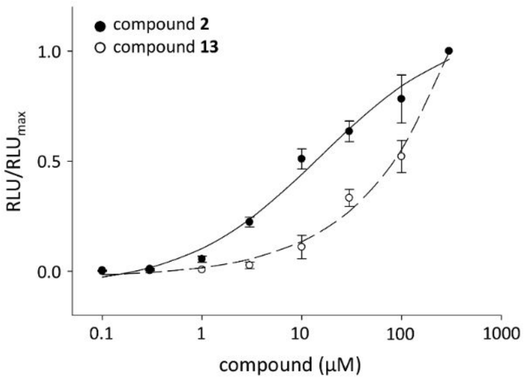

Figure S1. Concentration-response relations of 2-ethyl-3,6-dimethylpyrazine (compound 2) and 2-ethyl-3,5-dimethylpyrazine (compound 13) on OR5K1. Data were mock control-subtracted, normalized to the OR5K1 signal of each ligand, and displayed as mean ± SD of independent transfection experiments (n = 4). RLU = relative luminescence units.

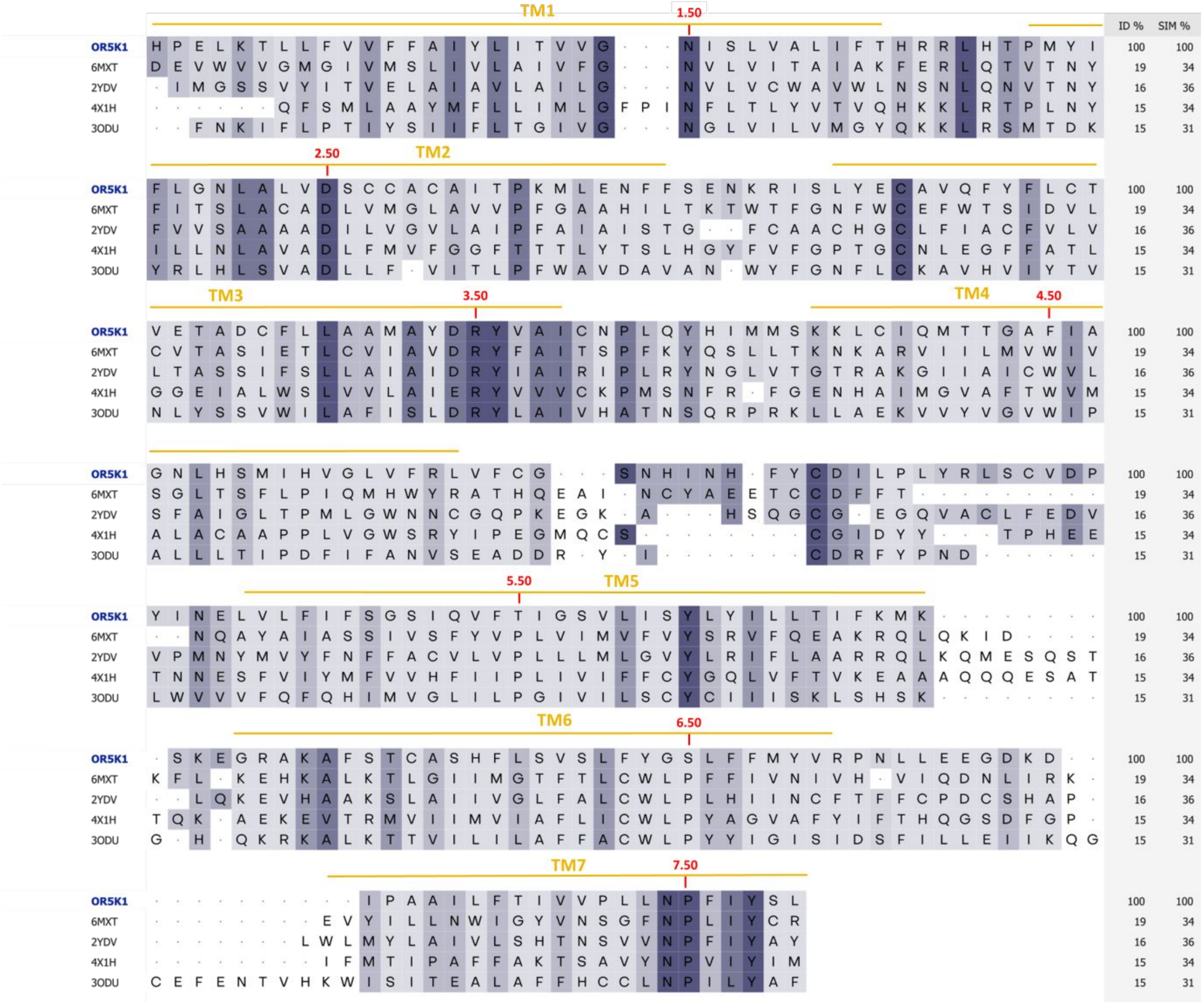

Figure S2. Multiple Sequence Alignment of OR5K1 (residues 20-292) to β2-adrenergic receptor (PDB ID: 6MXT), A2A receptor (PDB ID: 2YDV), Rhodopsin receptor (PDB ID: 4X1H), and CXCR4 receptor (PDB ID: 3ODU). Transmembrane (TM) domains and conserved BW positions are annotated. Template selection and sequence alignment from de March et al. 2015.(de March, Kim et al. 2015).

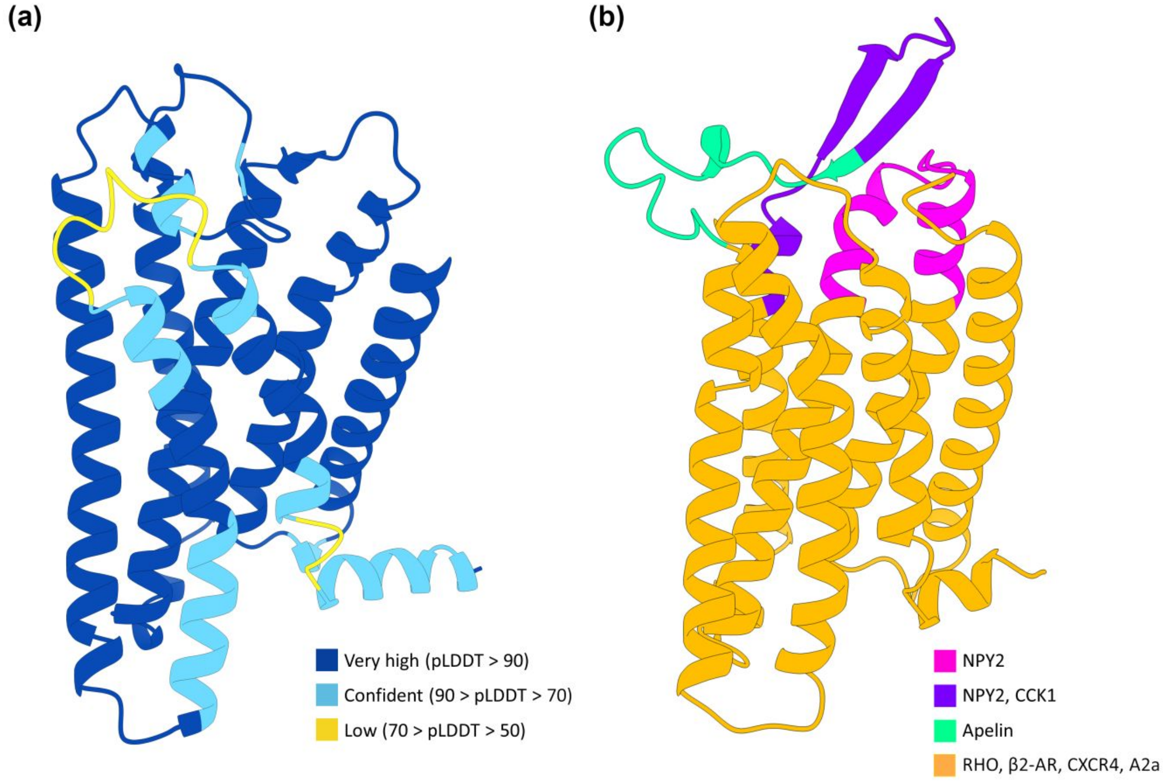

Figure S3. OR5K1 models built with AlphaFold 2 (a) and homology modeling (b). Alphafold 2 model (AF2) is colored by pLDDT confidence score, while OR5K1 model predicted with homology model (HM) is colored by templates coverage.

(a)

(b)

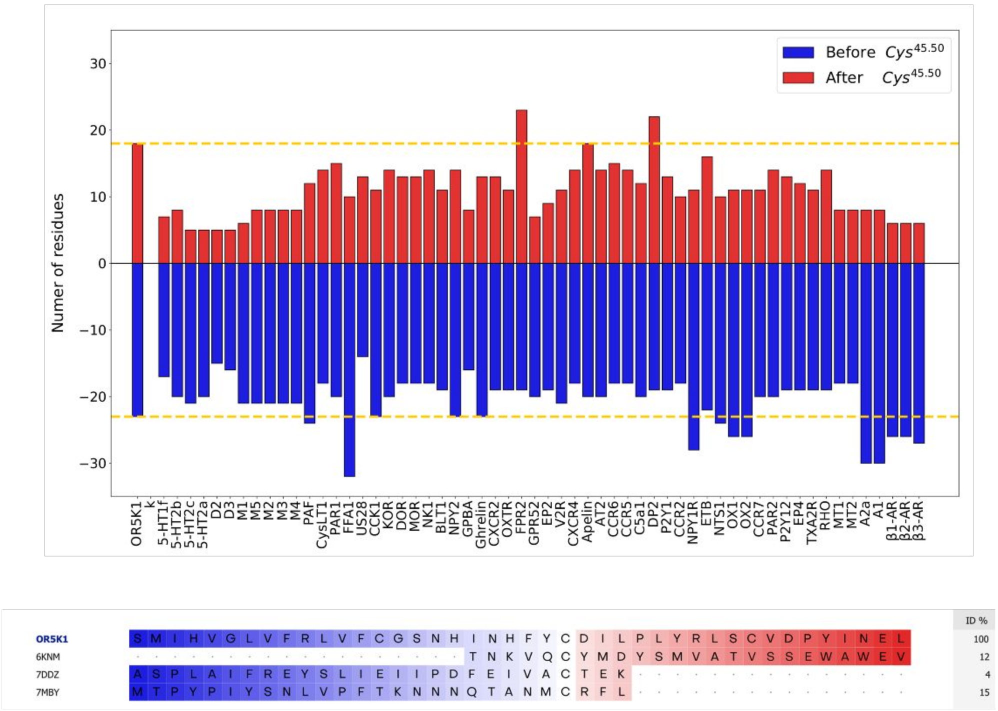

Figure S4. **(a)** Length of ECL2 for OR5K1 and experimental class A GPCRs. (**b)** Sequence Alignment of OR5K1 ECL2 region (S156$^{4.57}$- L197$^{5.37}$) to templates used for its modeling: NPY2 (PDB ID: 7DDZ) and CCK1 (PDB ID: 7MBY) before-Cys$^{45.50}$ segment and Apelin (PDB ID: 6KNM) after-Cys$^{45.50}$ segment.

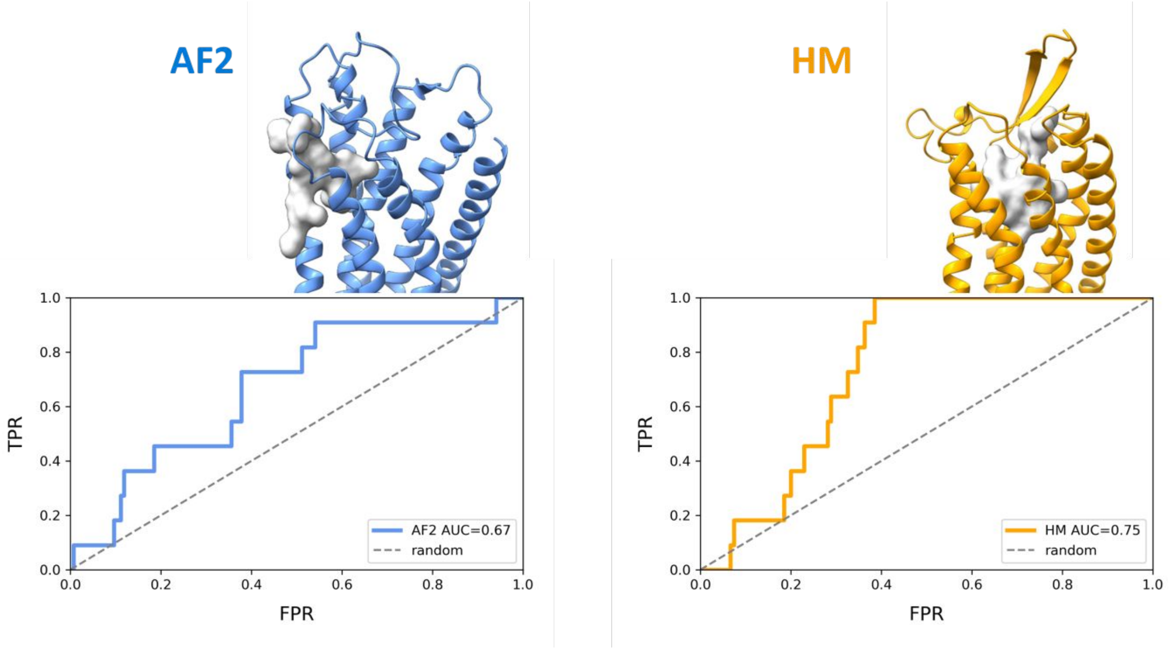

Figure S5. Binding site and ROC analysis of the starting OR5K1 AF2 and HM models. ROC curves plot the rate of true positives (TPR) against false positives (FPR). ROC curves are colored in blue for the AF2 model and orange for HM.

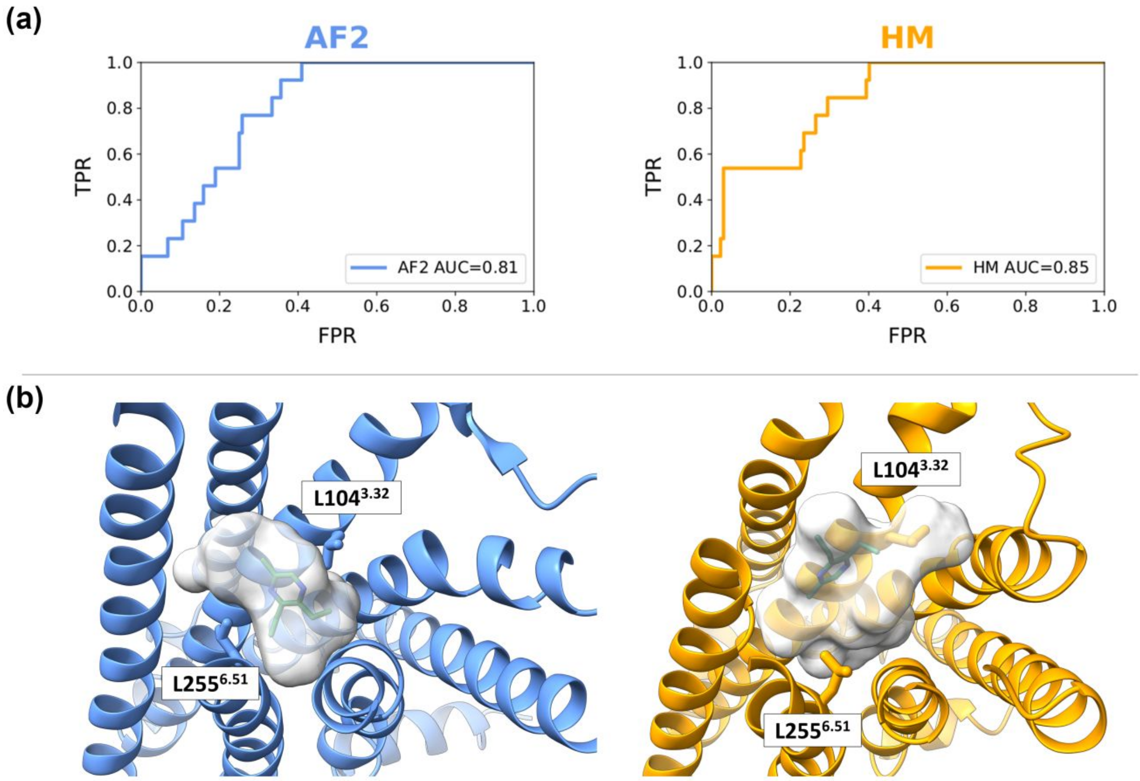

Figure S6. (a) ROC analysis of the OR5K1 AF2 and HM models after the first IFD simulation round. (**b)** Predicted binding modes of compound **1** within the OR5K1 binding site refined after the first IFD simulation round from AF2 and HM models.

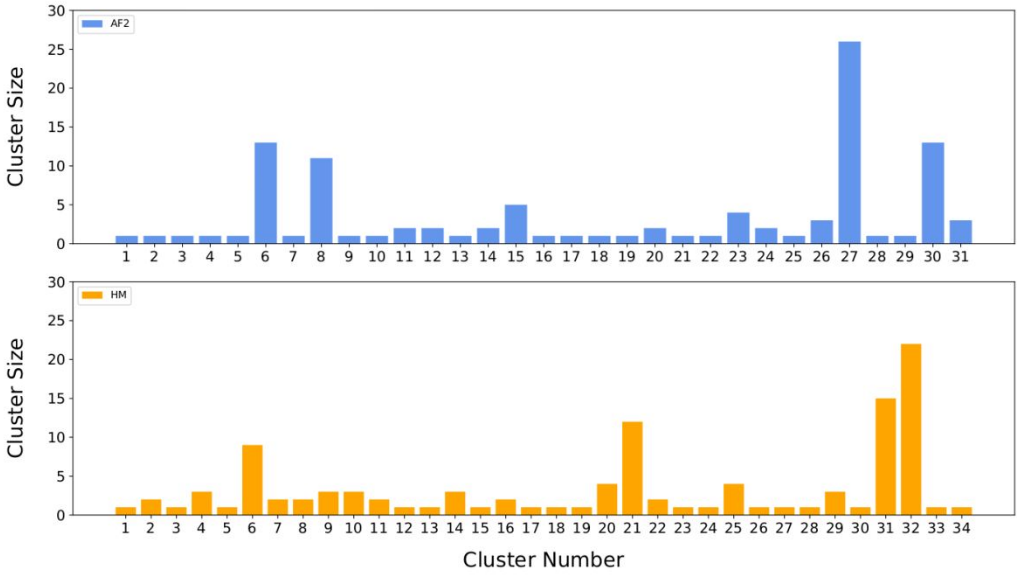

Figure S7. Distribution of the clusters binding poses of compound **1** in proximity to L104$^{3.32}$ and L255$^{6.51}$.

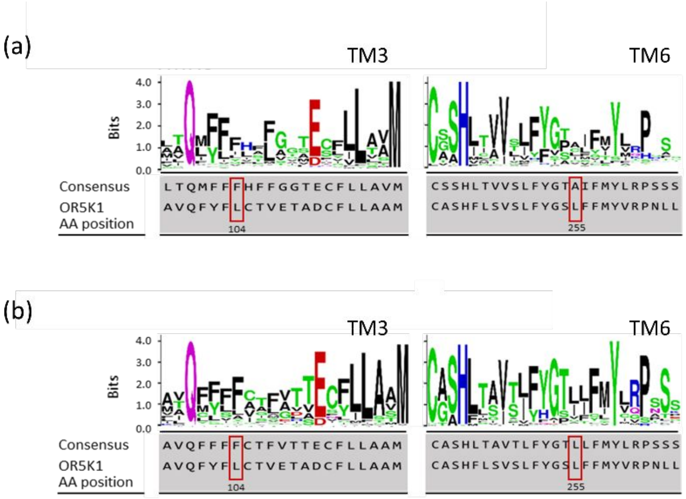

Figure S8. Leucine residues L104$^{3.32}$ and L255$^{6.51}$ are not conserved within all human and family 5 ORs.

Alignments of TM3 and TM6 of (a) human OR5K1 and the continuing 385 human ORs as well as (b) family 5 ORs. Shown are sequence logos, the consensus sequence, and the human OR5K1 sequence with the two leucine positions (red boxes). The consensus amino acid refers to the most frequent one, which is determined by letter height and stacking order. The letters of each stack are ordered from the most frequent to the least frequent. Amino acid conservation is measured in bits, and a 100% conservation correlates to 4.32 bits (Crooks, Hon et al. 2004). Basic amino acids (K, R, H) are blue, polar (G, S, T, Y C) are green, hydrophilic (Q, N) are purple, acidic (D, E) are red, and hydrophobic (A, V, L, I, P, W, M, F) are black.

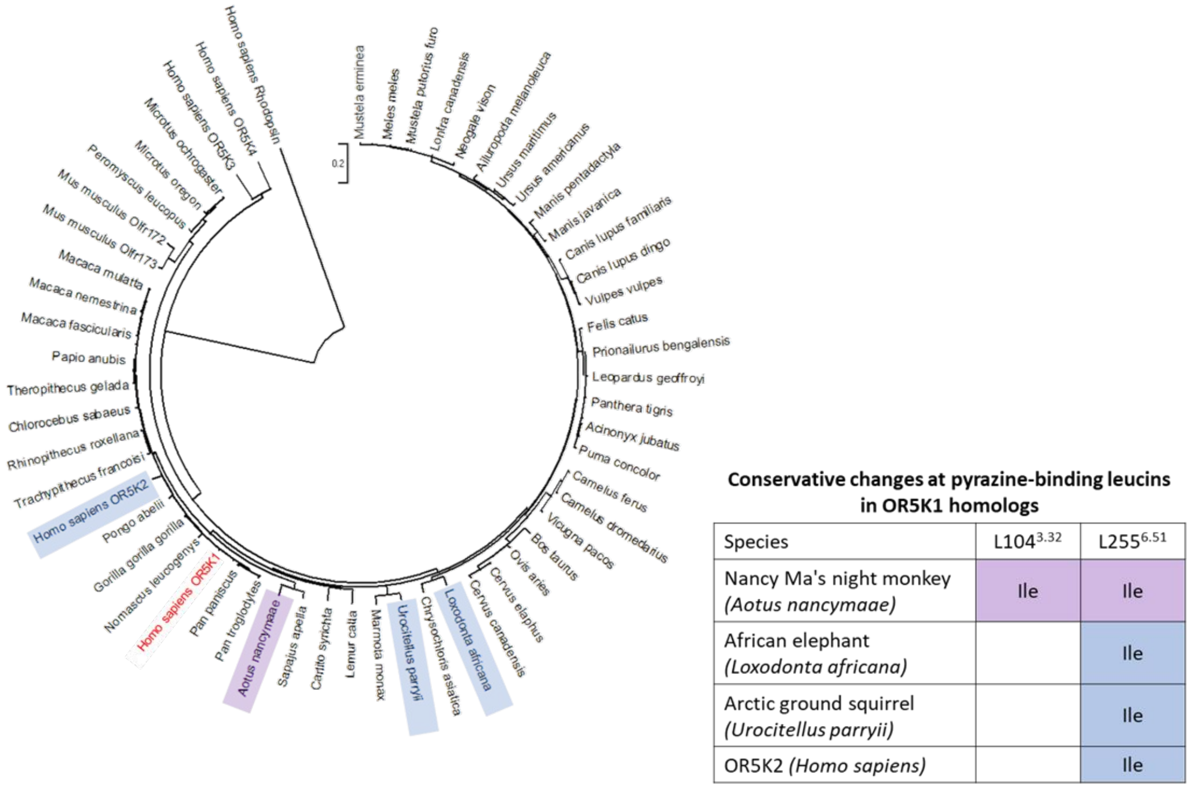

Conservative changes at pyrazine-binding leucins in OR5K1 homologs

Species	$L104^{3.32}$	$L255^{6.51}$
Nancy Ma's night monkey *(Aotus nancymaae)*	Ile	Ile
African elephant *(Loxodonta africana)*		Ile
Arctic ground squirrel *(Urocitellus parryii)*		Ile
OR5K2 *(Homo sapiens)*		Ile

Figure S9. Leucine residues $L104^{3.32}$ and $L255^{6.51}$ are highly conserved in OR5K1 homologs. Circular, phylogenetic relationship of OR5K1 homologs with human rhodopsin as outgroup. Marked in purple is the species with an amino acid exchange at both conserved leucines. Highlighted in blue are the species with an amino acid exchange at $L255^{6.51}$. Homo sapiens OR5K1 is highlighted in red.

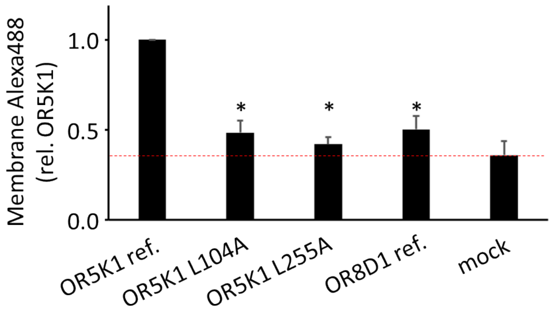

Figure S10. Cell-surface expression of OR5K1 and the two leucine mutants. Bar chart showing the relative surface expression of OR5K1, the two leucine mutants OR5K1 L104A and OR5K1 L255A as well as OR8D1 for comparison, using the flow cytometry assay. Red dashed line indicates the level of mock control. Data is displayed as mean ± SD (n = 3). Asterisks indicate statistically significant effects as compared to OR5K1 ref., identified by a paired t-test, with P < 0.05.

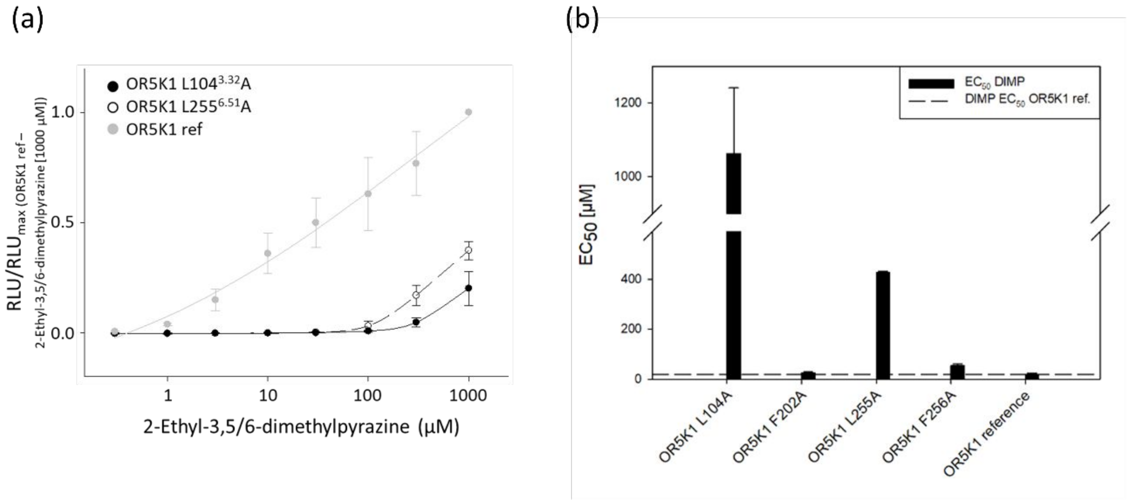

Figure S11. (a) Concentration−response relations of 2-ethyl-3,5/6-dimethylpyrazine on OR5K1 ref (grey circles), OR5K1 L104$^{3.32}$A (black circles), and OR5K1 L255$^{6.51}$A (white circles). Data were mock control-subtracted, normalized to the response of OR5K1 ref to 2-ethyl-3,5/6-dimethylpyrazine (1000 µM) and displayed as mean ± SD (n = 3). RLU = relative luminescence units. **(b)** Black boxes are DIMP EC_{50} values for mutants L104$^{3.32}$A, L255$^{6.51}$A, F202$^{5.42}$A, and F256$^{6.52}$A and wt OR5K1; dashed line represents

EC_{50} of the OR5K1 reference versus 2-ethyl-3,5/6-dimethylpyrazine (DIMP). All values were derived from at least n = 3 independent experiments measured via GloSensor™ technology.

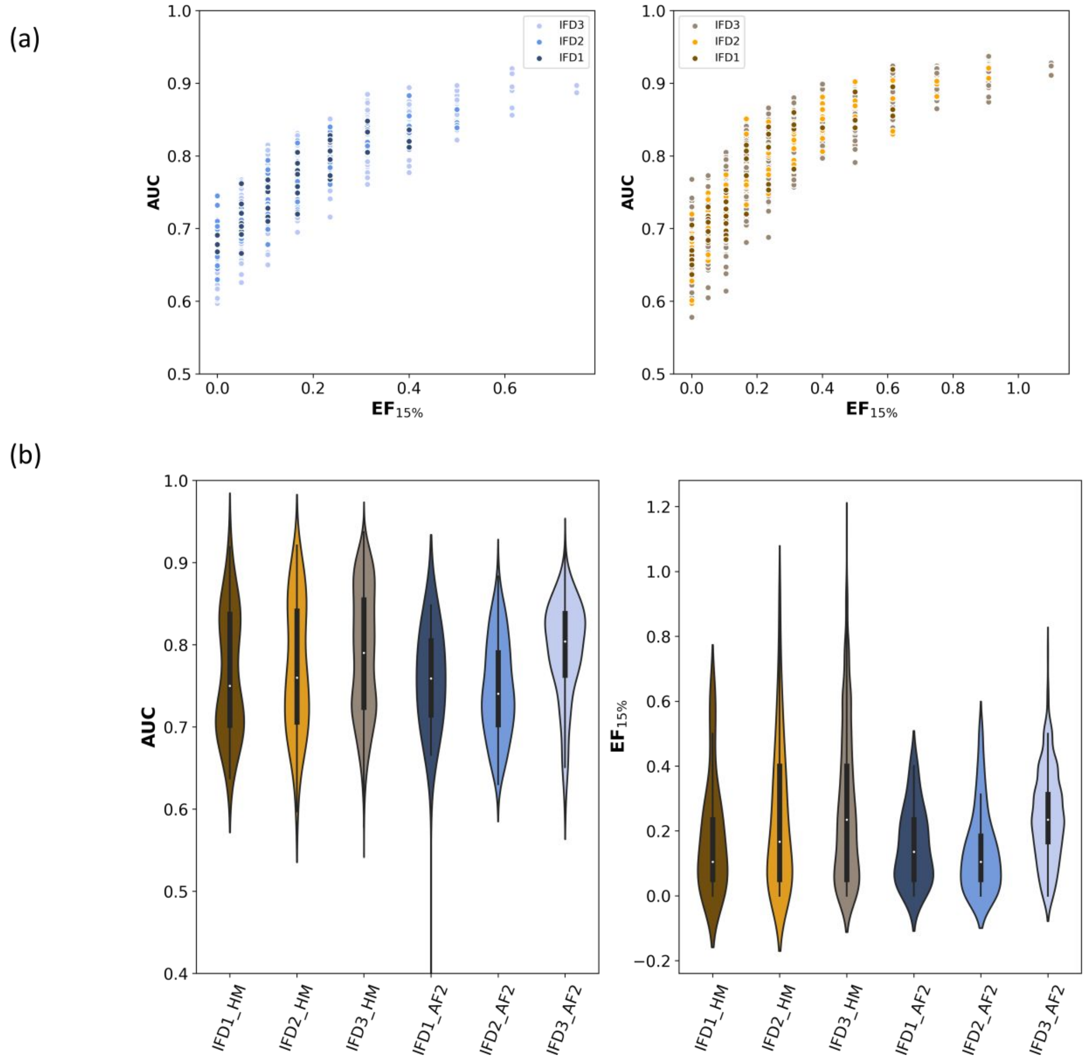

Figure S12. (a) Plots of EF vs. AUC values for the AF2 (blue shades) and HM models refined with IFD simulations **(b)** Distribution of AUC and EF values along the three rounds of IFD simulations.

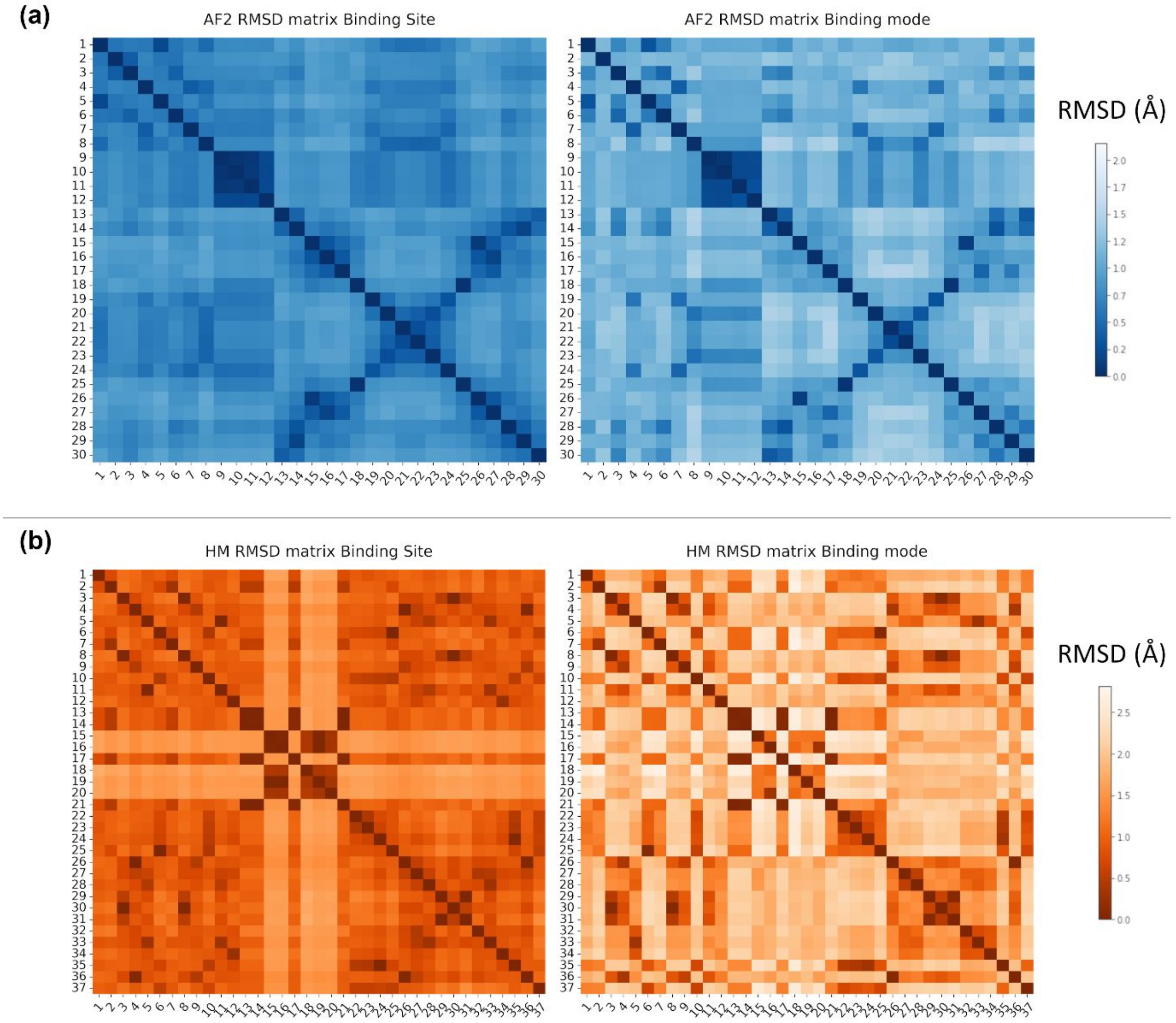

Figure S13. Heat maps representing the pairwise all atom RMSD matrices for the orthosteric binding site of the best performing models obtained after clustering IFD3 models. Matrices on the left refer to binding site residues, while matrices on the right include ligand coordinates in the selection of atoms for RMSD calculation. **(a)** Models from AF2. Cells are colored from dark orange to white according to increased RMSD values. Residues defining the binding site are: 104, 105, 108, 159, 199, 202, 206, 255, 256, 276, 279, 280. **(b)** Models from HM. Cells are colored from dark blue to white according to increased RMSD values. Residues defining the binding site are: 101, 104, 105, 108, 178, 180, 181, 199, 255, 258, 259, 275, 278, 279.

Models from IFD3 are available at https://github.com/dipizio/OR5K1_binding_site

Table S1. Models from IFD1 and IFD2 with d < 0.4, AUC > 0.8. Model names indicate if the model is resulting from the refinement of HM or AF2 models and if it is resulting from the first (IFD1) or second (IFD2) simulation round. We report AUC and $EF_{15\%}$ values, as well as information about the clustering and predicted van der Waals (vdW, kcal/mol) contribution of L104 and L255 to the binding of compound **1.** Models from IFD1 and IFD2 are available at https://github.com/dipizio/OR5K1_binding_site.

Model	AUC	$EF_{15\%}$	d (nm)	Cluster	Cluster Size	vdW L104$^{3.32}$	vdW L255$^{6.51}$
HM_IFD2_36	0.901	0.615	0.278	4	3	-2.801	-1.379
HM_IFD2_1	0.872	0.400	0.273	5	1	-2.477	-0.697
HM _IFD2_5	0.810	0.313	0.197	6	9	-3.376	-0.955
HM_IFD2_56	0.834	0.313	0.281	8	2	-2.295	-0.768
HM_IFD2_21	0.869	0.500	0.38	11	2	-1.58	-0.657
HM_IFD2_20	0.921	0.909	0.31	20	4	-0.329	-2.36
HM_IFD2_18	0.902	0.615	0.283	21	12	-1.698	-1.562
HM_IFD2_23	0.864	0.400	0.391	23	1	-1.903	-0.385
HM_IFD1_14	0.914	0.615	0.345	31	15	-0.273	-1.439
HM_IFD1_3	0.864	0.615	0.33	32	22	-0.221	-1.734
HM_IFD1_11	0.837	0.211	0.26	33	1	-0.183	-2.274
HM_IFD1_13	0.855	0.615	0.399	34	1	-0.125	-1.503
AF2_IFD2_120	0.833	0.235	0.285	8	11	-2.477	-0.697
AF2_IFD2_105	0.883	0.538	0.356	15	5	-1.036	-1.246
AF2_IFD2_55	0.808	0.400	0.265	21	1	-2.690	-0.937
AF2_IFD2_79	0.855	0.400	0.153	27	26	-2.258	-2.669
AF2_IFD2_70	0.864	0.500	0.149	30	13	-2.815	-1.221
AF2_IFD2_98	0.841	0.313	0.216	31	3	-2.675	-1.284

Table S2. Oligonucleotides for molecular cloning of OR5K1.

Gene	Oligo-nucleotide	Restriction Site	TM (°C)		Sequence 5´→3´
OR5K1	al-359	EcoRI	58	fw	CTGT *GAATTC* **ATG** GCT GAA GAA AAT CAT ACC ATG AAA AAT GAG TTT ATC
	al-360	NotI	59	rv	CTGC *GCGGCCGC* **GTA** ATT TCA CAT GGA AGT TTT TGC TGC ATT TAA ATC

TM = melting temperature, fw = forward, rv = reverse. Italic letters highlight the restriction sites. Start and Stop codons are printed in bold.

Table S3. Vector internal oligonucleotides.

Vector	Oligonucleotide	TM (°C)		Sequence 5´→3´
pFN210A	520	60	fw	GTG GAC ATC GGC CCG GGT C
	550	52	rv	CAC AAA TAA AGC ATT TTT TTC ACT GC

TM = melting temperature, fw = forward, rv = reverse

Table S4. Oligonucleotides for Homo sapiens OR5K1 site directed mutagenesis.

Gene	Oligonucleotide	TM (°C)		Sequence 5´→3´
OR5K1 L104A	pm-275	57	fw	*GTACAGTTTTATTTTGCTTGCACTGTGG*
	pm-276	57	rv	*GTTTCCACAGTGCAAGCAAAATAAAACTG*
OR5K1 L255A	pm-281	58	fw	*CATTATTCTATGGATCTGCTTTCTTCATGTAC*
	pm-282	58	rv	*GTACATGAAGAAAGCAGATCCATAGAATAATG*

TM = melting temperature, fw = forward, rv = reverse

Table S5. NCBI reference sequences of olfactory receptor genes investigated.

Gene Description	Species	Common Species Name	NCBI Reference Sequence (Accession-number)
OR5K1	*Acinonyx jubatus*	Cheetah	XP_014936391.1
OR5K1	*Ailuropoda melanoleuca*	Giant panda	XP_011231001.2
OR5K1	*Aotus nancymaae*	Nancy Ma's night monkey	XP_012332612.1
OR5K1	*Bos taurus*	Cattle	NP_001377368.1
OR5K1	*Camelus dromedarius*	Dromedary	XP_010976859.1
OR5K1	*Camelus ferus*	Wild Bactrian camel	XP_006181138.2

OR5K1	*Canis lupus dingo*	Australian dingo	XP_025272970.1
OR5K1	*Canis lupus familiaris*	Domestic dog	NP_001376061.1
OR5K1	*Carlito syrichta*	Philippine tarsier	XP_008064628.1
OR5K1	*Cervus canadensis*	Elk	XP_043304847.1
OR5K1	*Cervus elaphus*	Red deer	XP_043748867.1
OR5K1	*Chlorocebus sabaeus*	Green monkey	XP_007984292.2
OR5K1-like	*Chrysochloris asiatica*	Cape golden mole	XP_006868660.1
OR5K1	*Felis catus*	Domestic cat	XP_003991608.3
OR5K1	*Gorilla gorilla gorilla*	Western lowland gorilla	XP_004036007.1
OR5K1	*Homo sapiens*	Human	NP_001004736.2
OR5K2	*Homo sapiens*	Human	NP_001004737.1
OR5K3	*Homo sapiens*	Human	NP_001005516.1
OR5K4	*Homo sapiens*	Human	NP_001005517.1
OR5K1	*Lemur catta*	Ring-tailed lemur	XP_045396378.1
OR5K1	*Leopardus geoffroyi*	Geoffroy's cat	XP_045293055.1
OR5K1	*Lontra canadensis*	North American river otter	XP_032708517.1
OR5K1	*Loxodonta africana*	African bush elephant	XP_003418985.1
OR5K1	*Macaca fascicularis*	Crab-eating macaque	XP_045241289.1
OR5K1	*Macaca mulatta*	Rhesus macaque	NP_001180719.3
OR5K1	*Macaca nemestrina*	Southern pig-tailed macaque	XP_011732248.1
OR5K1	*Manis javanica*	Sunda pangolin	XP_036864239.1
OR5K1	*Manis pentadactyla*	Chinese pangolin	XP_036772454.1
OR5K1	*Marmota monax*	Groundhog	XP_046311290.1
OR5K1	*Meles meles*	European badger	XP_045859641.1
OR5K1	*Microtus ochrogaster*	Prairie vole	XP_005344986.1
OR5K1	*Microtus oregon*	Stoat	XP_041528659.1
Olfr172	*Mus musculus*	Mouse	NP_667212.2
Olfr173	*Mus musculus*	Mouse	NP_667211.2
OR5K1	*Mustela erminea*	Ermine	XP_032200493.1
OR5K1	*Mustela putoriusfuro*	Ferret	XP_004772048.1
OR5K1	*Neogale vison*	American mink	XP_044110727.1
OR5K1	*Nomascus leucogenys*	Northern white-cheeked gibbon	XP_003261757.2
OR5K1	*Ovis aries*	Domestic sheep	XP_004002914.1
OR5K1	*Pan paniscus*	Bonobo	XP_003821943.1
OR5K1	*Panthera tigris*	Tiger	XP_007096003.2
OR5K1	*Pan troglodytes*	Common chimpanzee	XP_526253.2
OR5K1	*Papio anubis*	Olive baboon	XP_031518815.1
OR5K1	*Peromyscus leucopus*	White-footed mouse	XP_028723391.1
OR5K1	*Pongo abelii*	Sumatran orangutan	XP_002813420.2
OR5K1	*Prionailurus bengalensis*	Leopard cat	XP_043451993.1
OR5K1	*Puma concolor*	Cougar	XP_025769507.1
OR5K1	*Rhinopithecus roxellana*	Golden snub-nosed monkey	XP_010352038.2
OR5K1	*Sapajus apella*	Tufted capuchin	XP_032154801.1
OR5K1	*Theropithecus gelada*	Gelada	XP_025233483.1
OR5K1	*Trachypithecus francoisi*	François' langur	XP_033067425.1
OR5K1	*Urocitellus parryii*	Arctic ground squirrel	XP_026258216.1
OR5K1	*Ursus americanus*	American black bear	XP_045641565.1
OR5K1	*Ursus maritimus*	Polar bear	XP_008700260.1

OR5K1	*Vicugna pacos*	Alpaca	XP_006208125.1
OR5K1	*Vulpes vulpes*	Redfox	XP_025846410.1

References:

Crooks, G. E., G. Hon, J.-M. Chandonia and S. E. Brenner (2004). "WebLogo: a sequence logo generator." Genome Research **14**(6): 1188-1190.

de March, C. A., S. K. Kim, S. Antonczak, W. A. Goddard, 3rd and J. Golebiowski (2015). "G protein-coupled odorant receptors: From sequence to structure." Protein Sci **24**(9): 1543-1548.
